# Nitrogen Deficiency-induced Bacterial Community Shifts in Soybean Roots

**DOI:** 10.1264/jsme2.ME21004

**Published:** 2021-07-06

**Authors:** Wataru Yazaki, Tomohisa Shimasaki, Yuichi Aoki, Sachiko Masuda, Arisa Shibata, Wataru Suda, Ken Shirasu, Kazufumi Yazaki, Akifumi Sugiyama

**Affiliations:** 1 Research Institute for Sustainable Humanosphere, Kyoto University, Uji, Japan; 2 Tohoku Medical Megabank Organization, Tohoku University, Sendai Japan; 3 Plant Immunity Research Group, RIKEN Center for Sustainable Resource Science, 1–7–22 Suehiro-cho. Tsurumi-ku, Yokohama, Kanagawa 230–0045, Japan; 4 Laboratory for Microbiome Sciences, RIKEN Center for Integrative Medical Sciences, Yokohama 230–0045, Japan

**Keywords:** nitrogen deficiency, soybean (*Glycine max*), *Methylobacteriaceae*, root microbiota

## Abstract

Nitrogen deficiency affects soybean growth and physiology, such as symbiosis with rhizobia; however, its effects on the bacterial composition of the soybean root microbiota remain unclear. A bacterial community analysis by 16S rRNA gene amplicon sequencing showed nitrogen deficiency-induced bacterial community shifts in soybean roots with the marked enrichment of *Methylobacteriaceae*. The abundance of *Methylobacteriaceae* was low in the roots of field-grown soybean without symptoms of nitrogen deficiency. Although *Methylobacteriaceae* isolated from soybean roots under nitrogen deficiency did not promote growth or nodulation when inoculated into soybean roots, these results indicate that the enrichment of *Methylobacteriaceae* in soybean roots is triggered by nitrogen-deficiency stress.

Nitrogen is an important mineral nutrient for crop production. Although the usage of synthetic nitrogen fertilizers has increased food production over the last century, a large amount of fossil fuels is used during their production and nitrogen leaching from agricultural soils causes environmental pollution (Stevens, 2019). Therefore, the development of alternative systems is urgently required to reduce the use of synthetic nitrogen fertilizers in crop production. Nitrogen affects the community composition of the root microbiota (Yeoh *et al.*, 2016; Konishi *et al.*, 2017; Chen *et al.*, 2019), which significantly impacts plant growth and immunity (Trivedi *et al.*, 2020). Heavy nitrogen fertilization often reduces the diversity of the root microbiota (Dai *et al.*, 2018; Xu *et al.*, 2020), and suppresses the colonization efficiency of diazotrophic bacterial species (Streeter and Wong, 1988; Naher *et al.*, 2018; Bahulikar *et al.*, 2021). A large-scale comparative analysis of the root microbiota of rice revealed that *NRT1.1B*, which encodes a rice nitrogen transporter and sensor, is a key gene that affects the community composition of the microbiota of field-grown rice (Zhang *et al.*, 2019). These findings imply that nitrogen is an important mediator of the interaction between hosts and their root microbiota; therefore, a more detailed understanding of nitrogen-dependent interactions is essential for maximizing the function of the root microbiota for sustainable crop production (Toju *et al.*, 2018).

Soybean (*Glycine max*) is one of the most important legume crops and establishes a symbiotic relationship with certain rhizobia, which are predominantly influenced by the supply of nitrogen. A heavy nitrogen supply suppresses nodule formation (Streeter and Wong, 1988), whereas nitrogen deficiency induces the secretion of the isoflavones, daidzein and genistein, which function as a signal for nodulation (Sugiyama *et al.*, 2016). We recently demonstrated that daidzein shifted the bacterial community composition in soil (Okutani *et al.*, 2020), suggesting that soybean modulates its root and rhizosphere microbiota by altering root exudation profiles in response to nitrogen deficiency. In addition, some bacterial members inhabiting soybean roots promote rhizobial growth and increase the efficiency of nodule formation (Cagide *et al.*, 2018; Han *et al.*, 2020; Zeffa *et al.*, 2020). These findings indicate the involvement of nitrogen nutrition and the root microbiota in rhizobial symbiosis and soybean growth; however, limited information is currently available on the effects of nitrogen availability on the root microbiota of soybean and its function. In the present study, we investigated the effects of nitrogen availability on the bacterial community composition of soybean roots.

To assess the effects of nitrogen availability on the bacterial community composition of soybean roots, we grew soybean (cv. Enrei) under nitrogen-sufficient and -deficient conditions. Soybean seeds were surface-sterilized with 70% ethanol and 1% sodium hypochlorite (NaClO) and germinated on sterile paper. After 5 days, seedlings were transferred to plastic pots (7.0‍ ‍cm diameter×9.0‍ ‍cm height) filled with vermiculite. Approximately 3.5‍ ‍g soil, collected from a soybean field of gray lowland soil without pesticide application at the Kyoto University of Advanced Science, Kameoka, Kyoto, Japan (34°99′38″N, 135°55′14″E), was placed on the surface of vermiculite as a bacterial inoculation source (hereafter designated as ‘soil’). Soil chemical properties were analyzed at the Tokachi Federation of Agricultural Cooperatives: pH of 7.1, 0.27% total N, 3.4% total C, 5.8% humin, 12.3 ppm NO_3_^–^, 529 ppm P, 1,008 ppm K, 3,648 ppm Ca, and 805 ppm Mg. Plants were grown in a culture room at 28°C under a light/dark (16/8 h) photoperiod and fertilized weekly with 50‍ ‍mL 1/2 nitrogen-free medium (Data Files S1) supplemented with 0 or 10‍ ‍mM KNO_3_ (hereafter defined as nitrogen-deficient and -sufficient conditions, respectively) (Fig. S1A). Plant and soil samples were collected at the V3 stage (26 days after sowing) (Fehr and Caviness, 1977). Harvested soybean roots were washed with tap water and nodules were removed from the roots by handpicking and then stored at –‍80°C until DNA extraction. The remaining root tissues were immediately subjected to bacterial isolation. In a community analysis of field-grown soybean, bulk, rhizosphere, and rhizoplane soils as well as endosphere samples were collected as described by Lundberg *et al.* (2012) using plants grown as previously reported (Okutani *et al.*, 2020).

Bacterial community profiling was performed by 16S rRNA gene amplicon sequencing, as previously described (Shimasaki *et al.*, 2021). In brief, the V4 region of 16S rRNA genes was amplified and sequenced using the MiSeq platform (Illumina). The sequence data obtained were analyzed using the QIIME2 platform, Version 2020.2 (Bokulich *et al.*, 2018). Detailed methods and computational analyses are provided in Supplemental Methods.

Root-inhabiting bacterial strains were isolated from soybean roots grown under nitrogen-deficient conditions, corresponding to the roots subjected to community profiling experiments (Fig. S1B). Individual bacteria isolates were identified by 16S rRNA gene sequencing and a BLAST search using the DDBJ database. Phylogenetic trees were constructed by the neighbor-joining method using MEGA version 7.0 (Kumar *et al.*, 2016). The whole-genome sequencing of *Methylobacteriaceae* isolates and their assembly was performed as previously described (Shimasaki *et al.*, 2021). Briefly, reads obtained by the PacBio sequel II platform were assembled and annotated using HGAP4 via SMRTlink (v 8.0.0) and DFAST (Tanizawa *et al.*, 2018), respectively. The presence of genes associated with the utilization of methylamine and ureide as well as nitrogen fixation were assessed by a BLAST search (setting: *E*-value thread >1.0×10^–20^). Detailed methods for the isolation, identification, phylogenetic analysis, and whole-genome sequencing of bacterial isolates are available in Supplemental Methods.

Soybean seeds were surface-sterilized and germinated as described above. Germinated seedlings were sown in a plant box (AGC Techno Glass, 60×60×100‍ ‍mm) filled with sterilized vermiculite and watered with 1/2 nitrogen-free media. Each bacterial strain was cultured in growth media (Data Files S1) until strains reached the stationary phase at 28°C. Bacterial suspensions were prepared in sterilized water at a concentration of 1×10^7^ for a single inoculation and 2×10^7^‍ ‍CFU‍ ‍mL^–1^ for a dual inoculation. A one-milliliter bacterial suspension was inoculated into plants (Fig. S1C). The corresponding value of CFU for OD_600_=1.0 was measured for each bacterial strain. Sterilized water was used as the control. Plants were cultivated between November 13, 2019 and December 18, 2019 (at the V4 stage) in a greenhouse set at a minimum of 20°C. After harvesting, the fresh weight of shoots and leaf chlorophyll contents were measured (Porra *et al.*, 1989). Statistical analyses using the *t*-test and Dunnett’s test to compare plant growth were performed using R software version 4.0.1 (Ihaka and Gentleman, 1996).

Soybean growth was significantly promoted under nitrogen-supplemented conditions (Fig. S2A), whereas nodule formation was suppressed (Fig. S2B), indicating nitrogen availability effects on soybean growth and physiology. A principal component analysis (PCoA) using weighted UniFrac distances showed significant shifts in the bacterial community compositions of both soil and soybean roots that were dependent on nitrogen availability (PERMANOVA by adonis: *P*=2.5×10^–2^ and 2.6×10^–2^, respectively; Fig. 1A). However, the shift in the bacterial community was more significant in soil than in soybean roots (Fig. S3), indicating that community shifts in roots were regulated by plants. The enrichment of the bacterial phylum *Proteobacteria*, including bacteria orders *Betaproteobacteriales*, *Rhizobiales*, and *Sphingomonadales*, was significantly greater in soybean roots than in soil (Fig. 1B). Among the order *Rhizobiales*, the relative abundance of the bacterial family *Beijerinckiaceae* was markedly enriched under nitrogen-deficient conditions (Fig. S4A), accounting for approximately 8.8% of all bacterial sequences (Fig. 1C). The bacterial family *Beijerinckiaceae* is classified as the family *Methylobacteriaceae* in the NCBI taxonomy. Therefore, according to the NCBI database, the family *Beijerinckiaceae* was hereinafter referred to as the‍ ‍family *Methylobacteriaceae*. The enrichment of *Methylobacteriaceae* was not observed in soil (Fig. 1C). In contrast to *Methylobacteriaceae*, nitrogen deficiency depleted *Chitinophagales*, belonging to the bacterial phylum *Bacteroidetes*, in both soil and soybean roots (Fig. S4B), suggesting that the depletion of *Chitinophagales* was not driven by plants. Collectively, these results indicated that soybean modulated the bacterial community composition of its root microbiota in response to nitrogen availability, and these community shifts were largely characterized by the enrichment of *Methylobacteriaceae* under nitrogen-deficient conditions.

To characterize the family *Methylobacteriaceae*, we isolated 111 individual bacterial strains from soybean roots grown under nitrogen-deficient conditions. Among them, 13 isolates belonged to *Methylobacteriaceae*. Phylogenic characterization using nearly complete 16S rRNA gene sequences revealed that these isolates were classified into two groups, closely related to *Methylobacterium aquaticum* (classified into Group I) and *Methylorubrum extorquens* (classified into Group II), respectively (Fig. 2) (Green and Ardley, 2018). Further phylogenic analyses based on the V4 region of the 16S rRNA gene retrieved from ASVs, assigned to *Methylobacteriaceae*, and sequences from our *Methylobacterium* and *Methylorubrum* isolates showed that ASV01 and ASV02 represented *Methylobacterium* (Group II) and *Methylorubrum* (Group I), respectively (Fig. S5A). ASV01 and 02 were both predominant in soybean roots grown under nitrogen-deficient conditions (Fig. S5B), indicating that our isolates were identical to the bacterial strains enriched in soybean roots grown under nitrogen-deficient conditions. The relative abundance of *Methylobacteriaceae* was low in field-grown soybean without apparent symptoms of nitrogen deficiency, such as the yellowing of leaves (Fig S6), which is consistent with the results obtained from pot experiments.

Nitrogen fertilization often affects the colonization of diazotrophs (Streeter and Wong, 1988; Naher *et al.*, 2018; Bahulikar *et al.*, 2021) and *Methylobacterium nodulans* has been shown to nodulate with a particular leguminous plant and fix atmospheric nitrogen (Sy *et al.*, 2001). In addition, *Methylorubrum* has been identified as a predominant bacterial species in soybean stems and leaves over the growing stages (Hara *et al.*, 2019), and its colonization of above-ground tissues was also shown to be affected by a heavy nitrogen supply (Ikeda *et al.*, 2010, 2011). A metagenomic analysis of the family *Methylobacteriaceae* indicated that methylamine and ureide are potent nutrient sources for *Methylorubrum* inhabiting the above-ground tissues of soybean (Minami *et al.*, 2016). To assess the involvement of nitrogen fixation and the utilization ability of *Methylobacteriaceae* in their enrichment in the below-ground tissues of soybean, we performed the whole-genome sequencing of two representative isolates from each group (Fig. 2). *NifH* gene was not detected in *Methylobacterium* or *Methylorubrum* (Fig. S7A), suggesting that the enrichment of *Methylobacteriaceae* was not associated with nitrogen fixation ability. Methylamine and ureide utilization genes were identified in both *Methylorubrum* isolates (Fig. S7B and C). On the other hand, *Methylobacterium* sp. strain GmRoot51 possessed only some urea degradation genes and none of the methylamine or ureide utilization genes were detected in the genome of GmRoot62 (Fig. S7B and C). These results suggested that the methylamine and ureide catabolic capacity was not a prerequisite for the enrichment of *Methylobacterium*, whereas *Methylorubrum* may share a common biological process to adapt to both the above- and below-ground tissues of soybean.

Recent advances demonstrated that when plants are subjected to biotic and abiotic stress, they recruit particular bacterial species, which improve host fitness under the corresponding stress (Bakker *et al.*, 2018; Liu *et al.*, 2020). Furthermore, some bacterial species enhance the performance of rhizobial symbiosis, which is known as a ‘helper’ function. We examined the effects of a single inoculation and co-incubation with *Bradyrhizobium diazoefficiens* USDA110 on soybean growth and symbiosis with rhizobia under nitrogen-deficient conditions. Although *Methylorubrum* sp. strain GmRoot31 slightly increasing the shoot and nodule weights of soybean, no significant growth-promoting effects or helper functions were observed (Fig. 3 and S8).

In conclusion, the present study demonstrated nitrogen deficiency-induced community shifts in the soybean root microbiota. Although *Methylobacteriaceae* isolates from soybean roots did not promote soybean growth under nitrogen-deficient conditions, at least under our experimental conditions, several *Methylobacterium* spp. have been shown to exhibit plant growth-promoting activities towards a wide range of plant species, including soybean, rice, barley, and potato (Subramanian *et al.*, 2015; Tani *et al.*, 2015; Grossi *et al.*, 2020; Lai *et al.*, 2020). Root-derived bacteria are generally adapted to the host root environment (Bai *et al.*, 2015), which is beneficial for their role as bioinoculants in agricultural fields. Further studies on colonization mechanisms and functions are warranted to elucidate the ecological relevance and roles of *Methylobacteriaceae* in soybean roots under nitrogen-deficient conditions.

## Citation

Yazaki, W., Shimasaki, T., Aoki, Y., Masuda, S., Shibata, A., Suda, W., et al. (2021) Nitrogen Deficiency-induced Bacterial Community Shifts in Soybean Roots. *Microbes Environ ***36**: ME21004.

https://doi.org/10.1264/jsme2.ME21004

## Supplementary Material

Supplementary Material 1

Supplementary Material 2

## Figures and Tables

**Fig. 1.**
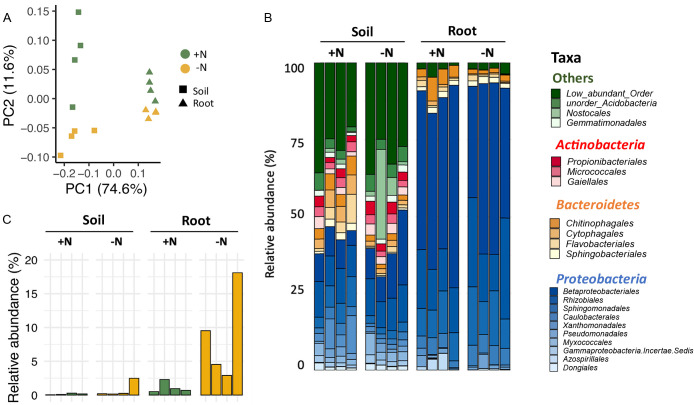
Bacterial community composition in the soybean root and soil under different nitrogen conditions. (A) PCoA based on weighted UniFrac distances. (B) Composition of the 20 most abundant bacterial orders across samples. (C) The relative abundance of *Methylobacteriaceae* in each sample.

**Fig. 2.**
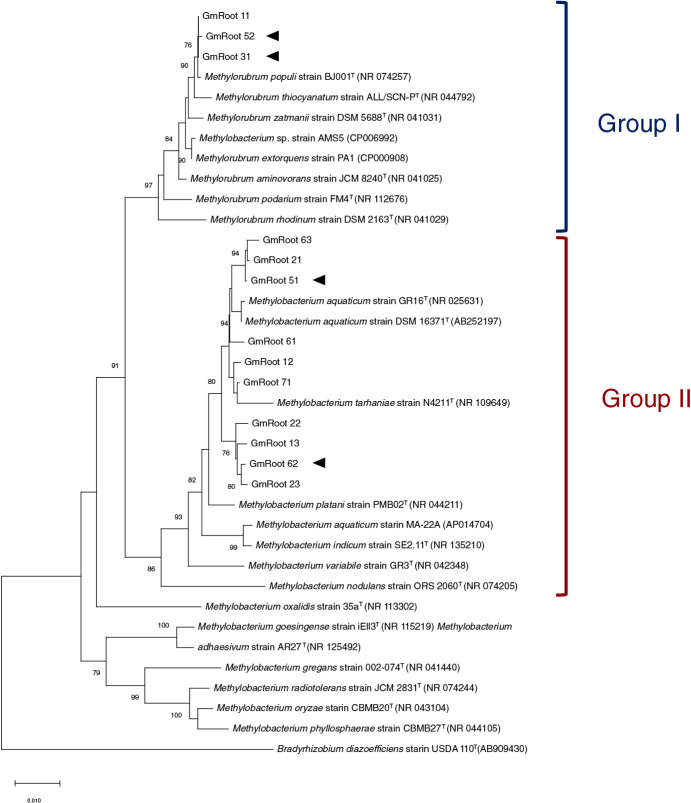
Neighbor-joining phylogenetic tree of *Methylobacteriaceae* isolates based on their near-complete 16S rRNA gene sequences. The phylogenetic tree was constructed by the neighbor-joining method. Bootstrap values (1,000 replicates) higher than 70% are shown in nodes. The isolates subjected to whole-genome sequencing and the inoculation test are marked with arrowheads. ^T^, Type strain.

**Fig. 3.**
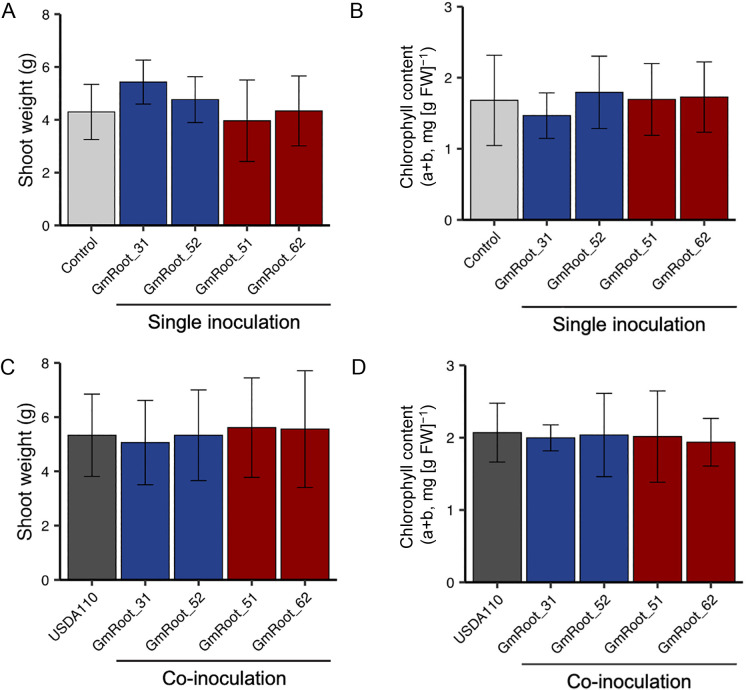
Effects of selected *Methylobacteriaceae* isolates on soybean growth. (A) Shoot weight and (B) chlorophyll content of soybean inoculated with a single *Methylobacteriaceae* isolate. (C) Shoot weight and (D) chlorophyll content of soybean co-inoculated with a selected *Methylobacteriaceae* isolate and *Bradyrhizobium diazoefficiens* USDA110. Error bars represent the standard deviation (*n*=6–8).
