# Supplementary material for: Nitrogen Deficiency-induced Bacterial Community Shifts in Soybean Roots
Source: Microbes Environ. 2021 Jul 6;36(3):ME21004. doi: 10.1264/jsme2.ME21004 (PMC8446753; doi:10.1264/jsme2.ME21004)
Supplement: Supplementary file 1 — Supplementary Material 1 [file 36_21004_s1.pdf]

Supplemental figures and tables

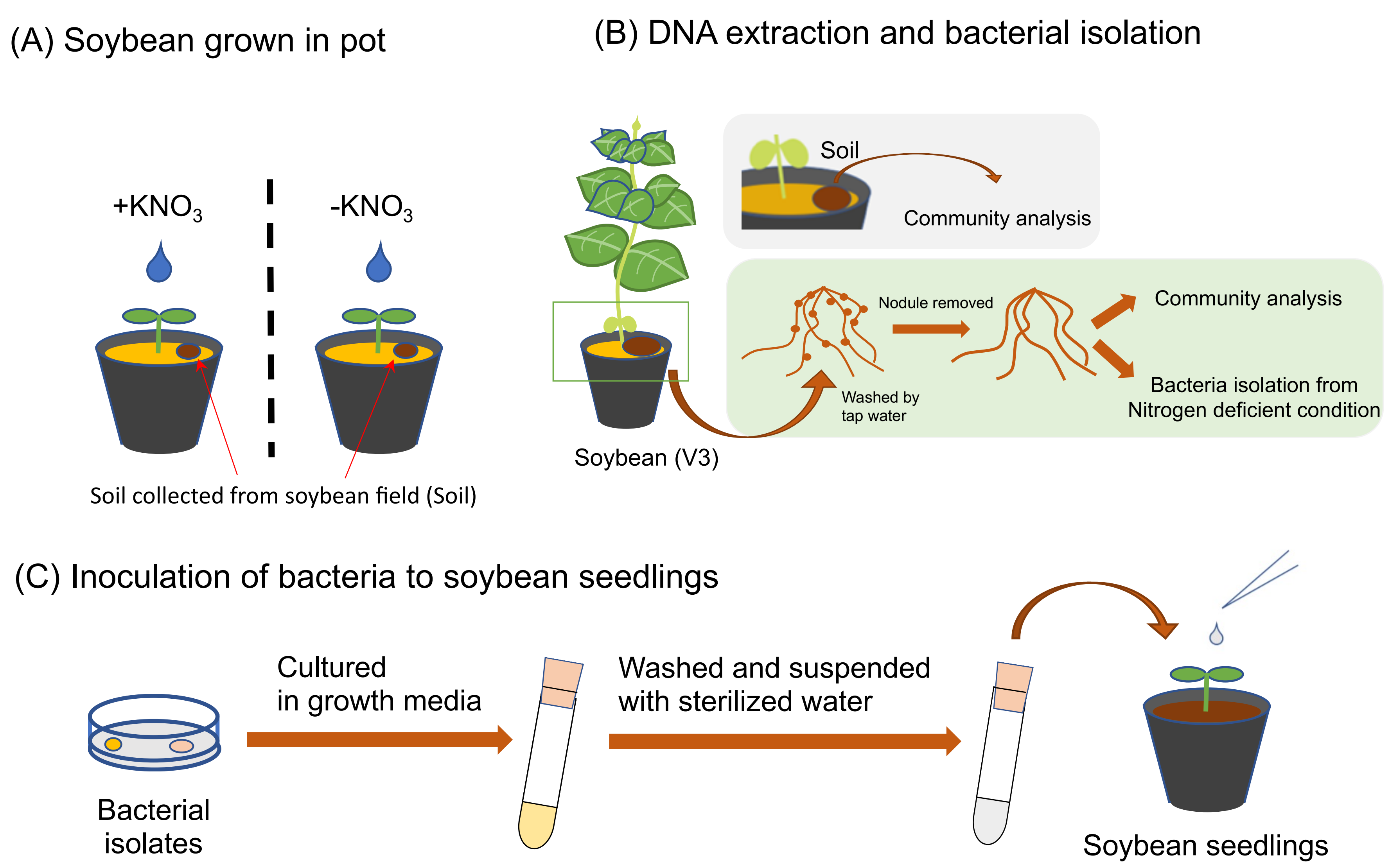

Fig. S1. Outline of experimental scheme.

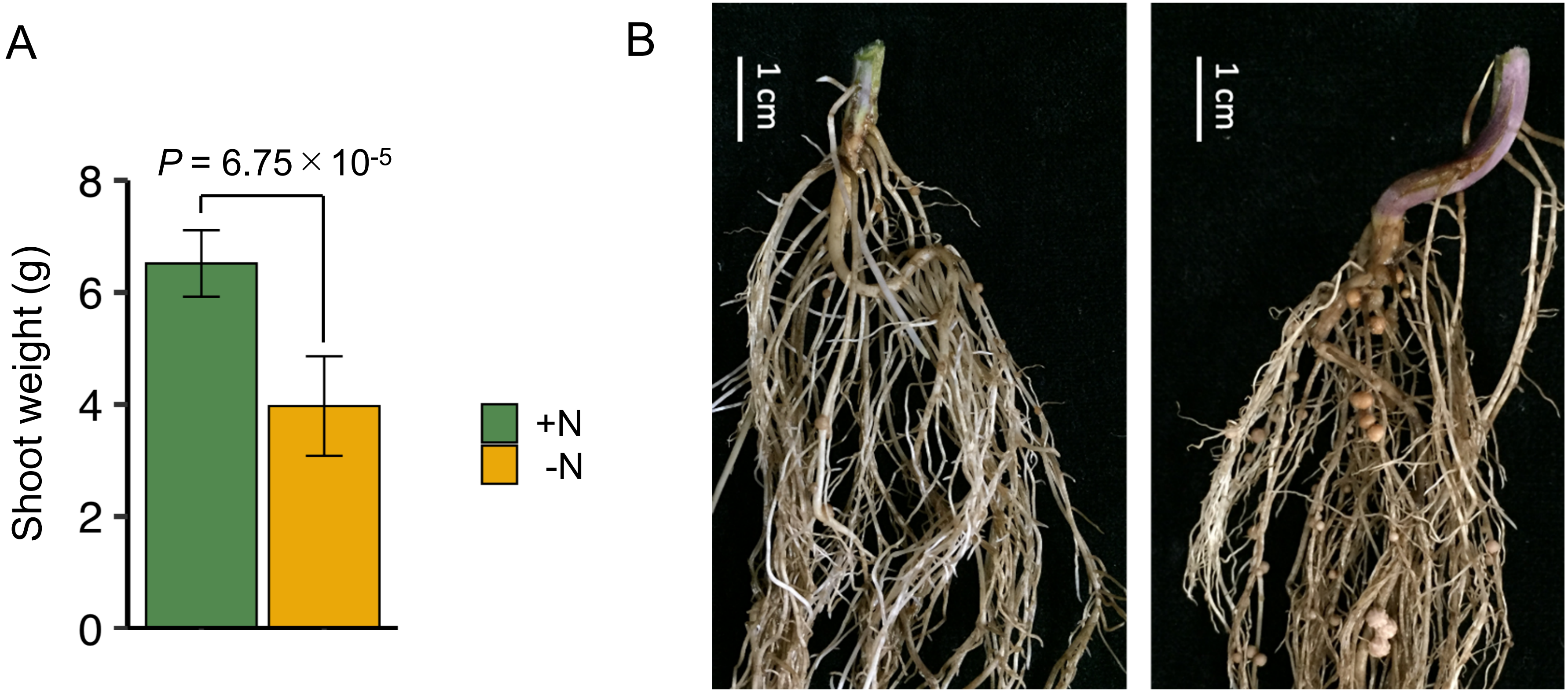

Fig. S2. Effect of nitrogen supply on soybean and nodulation of soybean.

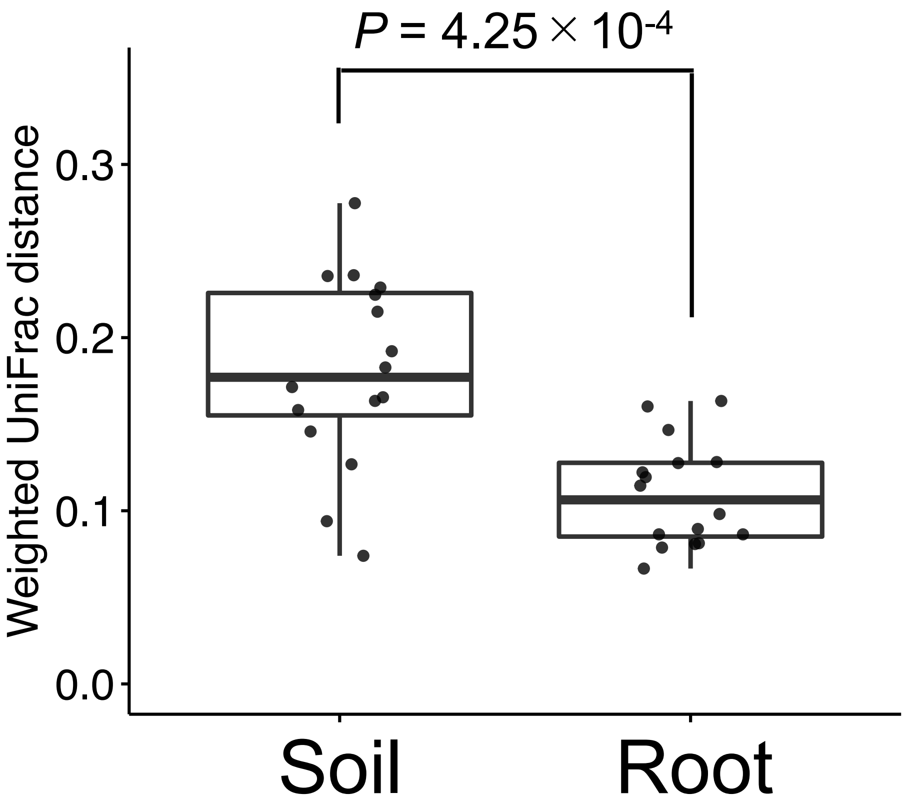

Fig. S3. Comparison of the extent of bacterial community shift in bulk soil and soybean roots.

Yazaki *et al.*,

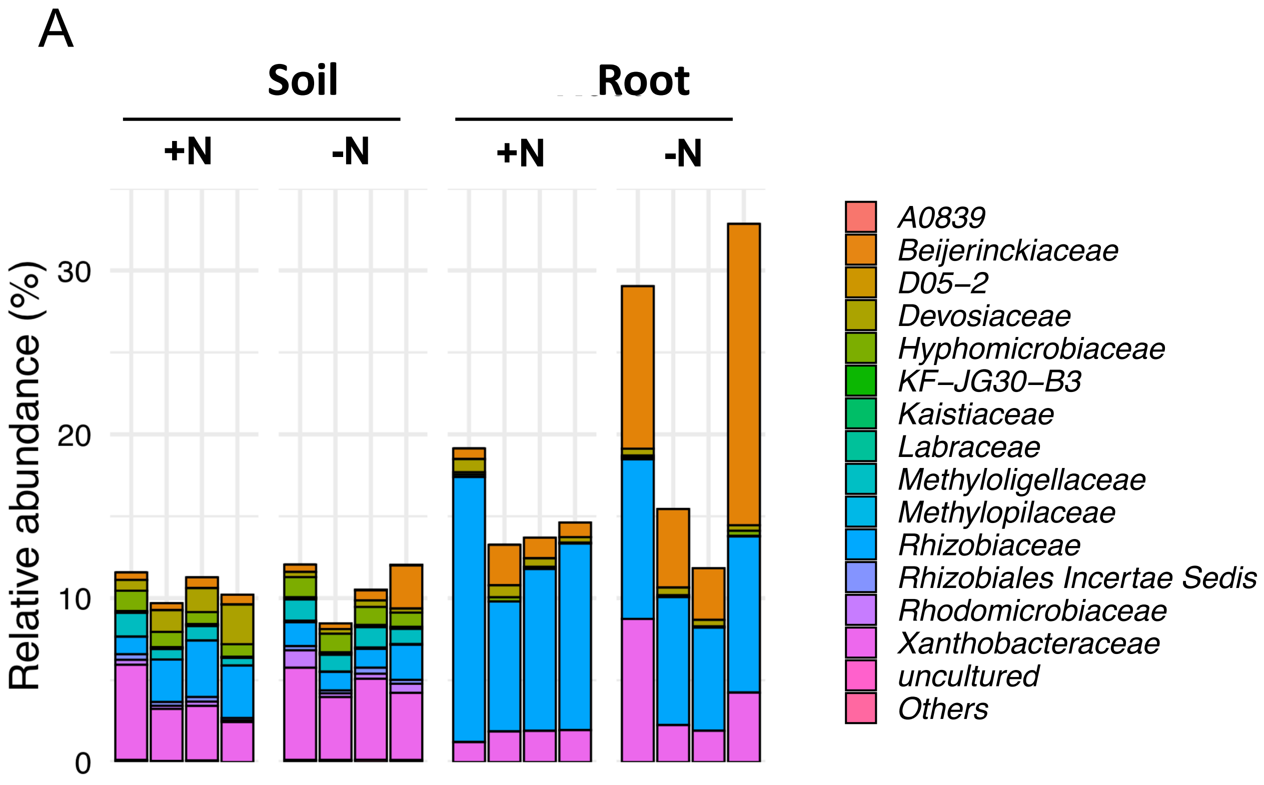

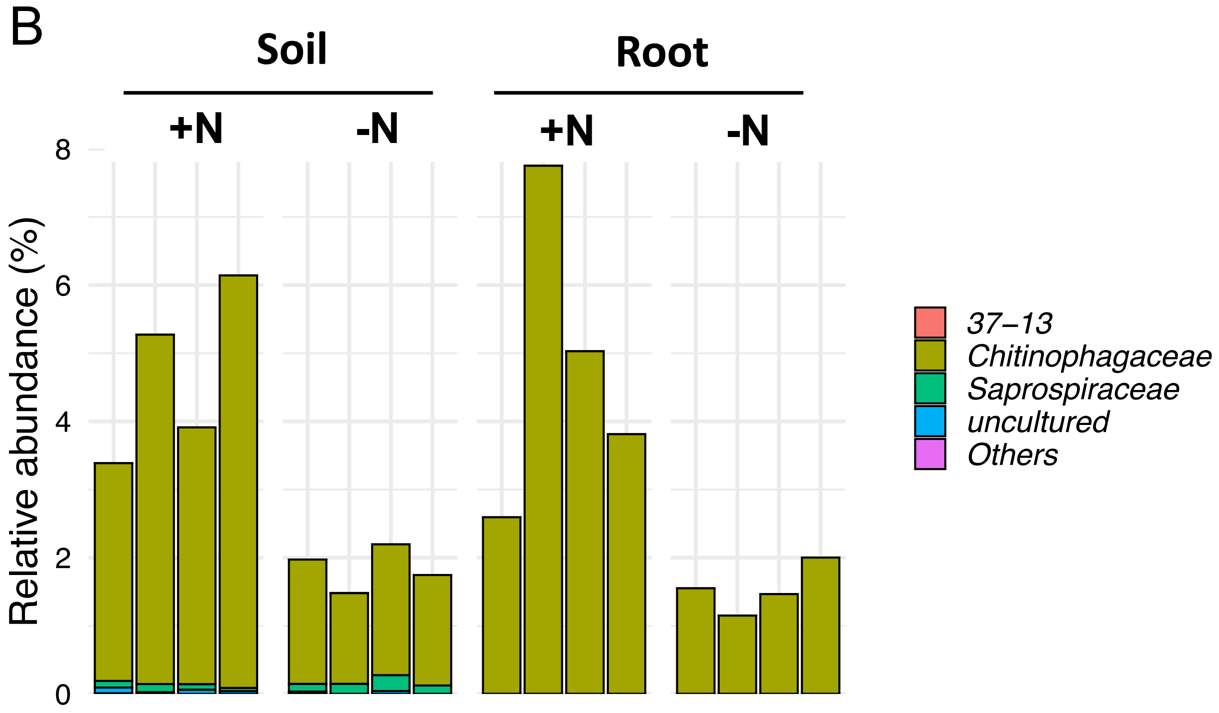

Fig. S4. Composition of bacterial community at family level.

Yazaki *et al.,*

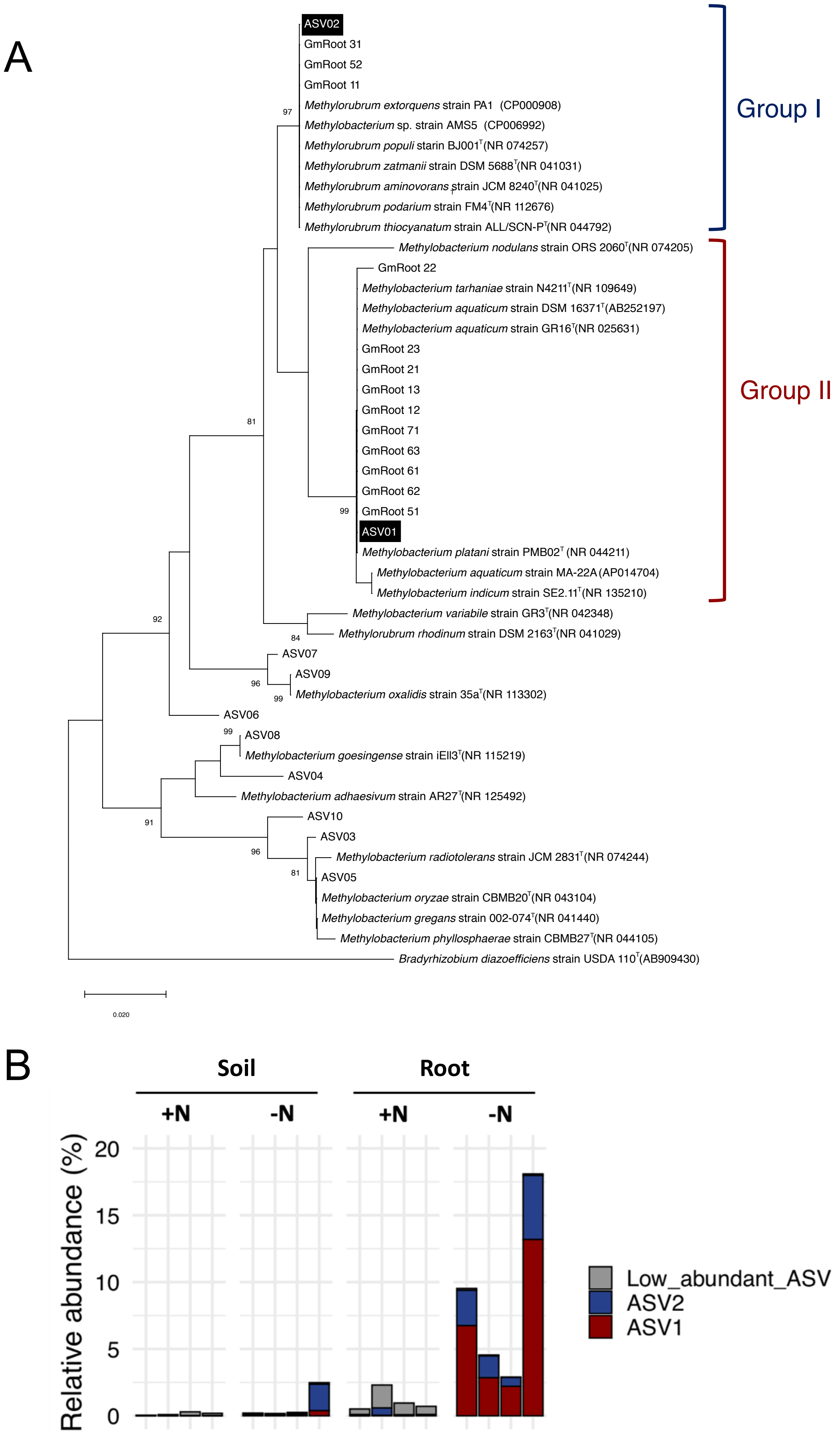

Fig. S5. ASV-level taxonomic composition of *Methylobacteriaceae*.

Yazaki *et al.*,

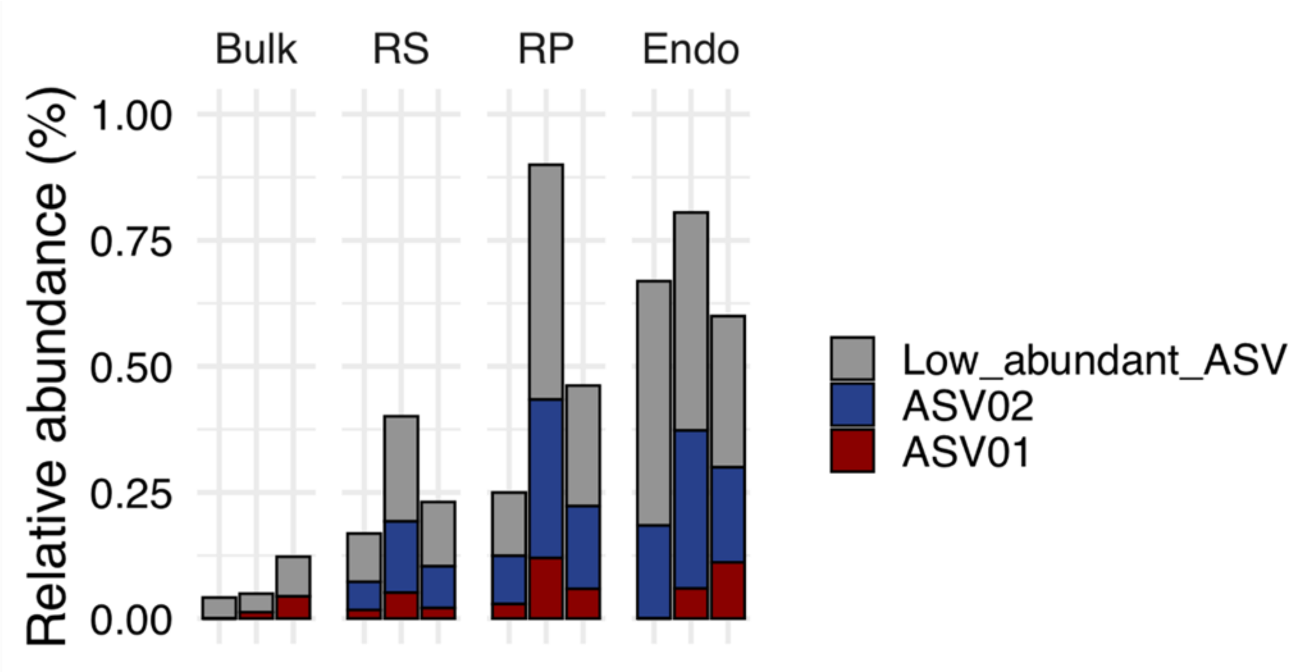

Fig. S6. Relative abundance of ASVs in field-grown soybean roots.

A Nitrogen fixation

Gene	Accession	*Methylorubrum* sp. GmRoot31	*Methylorubrum* sp. GmRoot52	*Methylobacterium* sp. GmRoot51	*Methylobacterium* sp. GmRoot62
nifH	CAD54428	○	○	○	○

B *N*-methylglutamate pathway

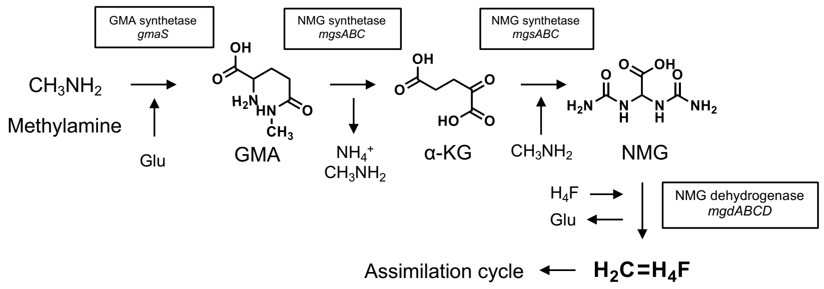

Gene	Genome region	*Methylorubrum* sp. GmRoot31	*Methylorubrum* sp. GmRoot52	*Methylobacterium* sp. GmRoot51	*Methylobacterium* sp. GmRoot62
gmasS	2224998-2226296	●	●	○	○
mgsA	2221941-2222876	●	●	○	○
mgsB	2222876-2223565	●	●	○	○
mgsC	2223565-2224908	●	●	○	○
mgdA	2219317-2220567	●	●	●	○
mgdB	2218912-2219199	●	●	○	○
mgdC	2215886-2218915	●	●	●	○
mgdD	2215264-2215893	●	●	○	○

C Allantonin and urea degradation pathway

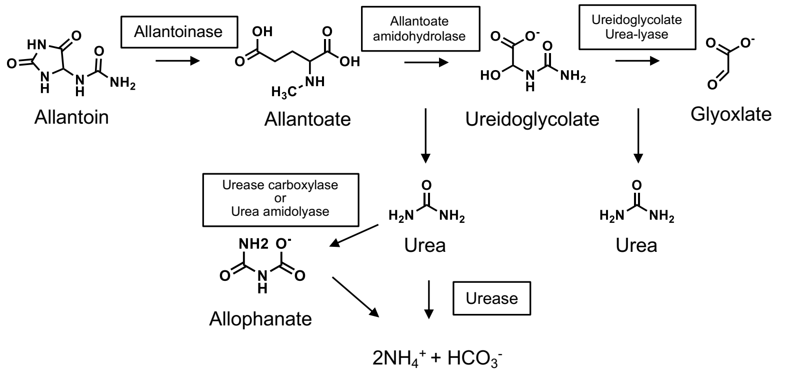

Gene	Genome region	*Methylorubrum* sp. GmRoot31	*Methylorubrum* sp. GmRoot52	*Methylobacterium* sp. GmRoot51	*Methylobacterium* sp. GmRoot62
Allantoinase	1770914-1769502	●	●	●	○
Allantoate amidohydrolase	3788200-3786950	●	●	●	○
Ureidoglycolate lyase	1765327-1764830	●	●	○	○
Urease	3161435-3163147	●	●	●	○
Urea amidolyase	1508397-1509416	●	●	○	○
Urea carboxylase	3980099-3980911	●	●	○	○

Presence ● / Absence ○ of gene

Fig. S7. Presence of nitrogen fixation and utilization related genes.

Yazaki *et al.,*

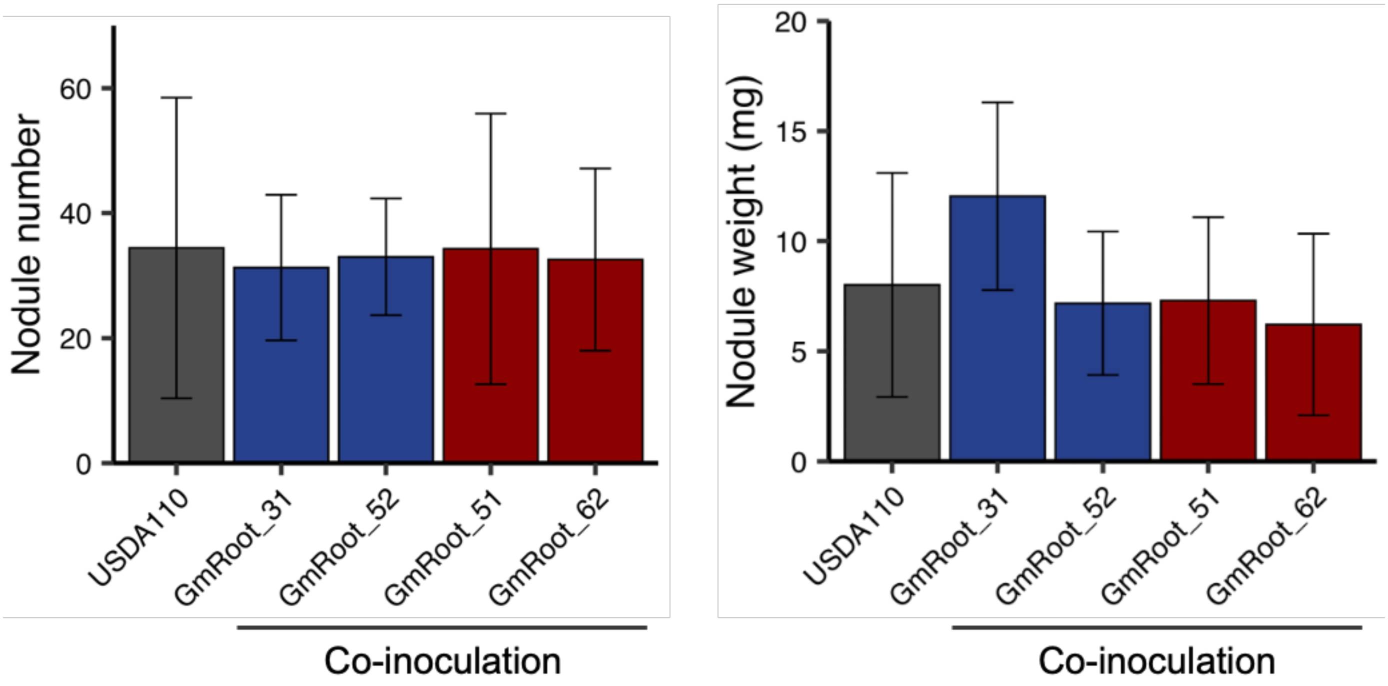

Fig. S8. Effect of selected Methylobacteriaceae isolates on rhizobia symbiosis.

Table S1. Primer and PNA sequences used in this study.

10F	5'-GTTTGATCCTGGCTCA-3'
800R	5'-TACCAGGGTATCTAATCC-3'
1500R	5'-TACCTTGTTACGACTT-3'
M13F	5'-GTAAAACGACGGCCAGT-3'
M13R	5'-CAGGAAACAGCTATGAC-3'
mPNA	N-term-GGCAAGTGTTCTTCGGA-C-term
pPNA	N-term-GGCTCAACCCTGGACAG-C-term

Supplementary materials

Fig. S1. Outline of experimental scheme. (A) Soybean were grown in nitrogen sufficient or deficient condition. **(B)** Bacterial community of soybean roots and soil were analyzed. Root-colonizing bacteria was isolated from soybean roots grown in nitrogen deficient condition. **(C)** The effect of *Methylobacteriaceae* isolates on soybean growth were tested by single and co-incubation with *B. diazoefficiens* USDA110.

Fig. S2. Effect of nitrogen supply and nodulation of soybean (A) Comparison of soybean shoot weight grown in nitrogen-deficient or -sufficient conditions. Error bars represent standard deviation (n = 7–8). **(B)** Photographs of nodules on soybean roots at sampling.

Fig. S3. Comparison of the extent of bacterial community shift in soil and soybean roots. Soil, weighted UniFrac distance between soil of nitrogen-deficient and -sufficient conditions; Root, weighted UniFrac distance between soybean roots grown in nitrogen-deficient and -sufficient conditions.

Fig. S4. Composition of bacterial community at family level. Composition of the bacterial family belonging to the order **(A)** *Rhizobiales* and **(B)** *Chitinophagales*.

Fig. S5. ASV-level taxonomic composition of *Methylobacteriaceae*. (A) Representative sequences of the ASVs belonging to *Methylobacteriaceae* were retrieved and used to infer phylogenetic

relationships with the strains isolated from roots using the neighbor-joining method. Bootstrap values (1000 replicates) above 70% are shown in nodes. ^{T}, Type strain. **(B)** The relative abundance of ASVs in each sample.

Fig. S6. Relative abundance of ASVs in field-grown soybean roots. Bulk, bulk soil; RS, rhizosphere; RP, rhizoplane; and ES, endosphere.

Fig. S7. Presence of nitrogen fixation and utilization related genes. The presence of genes associated with **(A)** nitrogen fixation, **(B)** *N*-methylglutamate pathway, and **(C)** allantonin and urea degradation pathway were surveyed by Blast search. Metabolic pathway of *N*-methylglutamate and ureide in *Methylobacteriaceae* are also shown in **(B)** and **(C)**, respectively. Query sequences of each pathway were retrieved from the genome sequence of *Methylobacterium nodulans* strain ORS 2060 (NC_011894), *Methylobacterium extorquens* strain DM4 (FP103042), and *Methylobacterium* sp. strain AMS5 (CP006992), respectively. The presence and absence of each gene is represented by closed and opened circles, respectively. GMA, γ-glutamylmethylamide; NMG, *N*-methylglutamate; α-KG, α-ketoglutarate; Glu, glutamic acid; H_4F, tetrahydrofolate.

Fig. S8. Effect of selected *Methylobacteriaceae* isolates on rhizobia symbiosis. (A) nodule weight and **(B)** Nodule number. Error bars represent standard deviation (n = 7–8).

Table S1. Primer and PNA sequences used in this study.

Data Files S1. Medium compositions used in this study and summary of *Methylobacteriaceae* isolates. Blast search of near-complete 16S rRNA gene sequences were performed using NCBI database.

Supplementary Methods

Supplemental Methods

Bacterial community analysis using 16S rRNA gene amplicon sequencing

Total DNA was extracted from the soil and plant samples using DNeasy PowerSoil Kit (Qiagen) according to the manufacturer's protocol. Extracted DNA was quantified using Qubit Quantification Platform dsDNA HS Assay Kit (Invitrogen). PCR amplification of the region V4 of 16S rRNA gene was performed with KOD Fx Neo Polymerase using first PCR primers 515F (5′-ACACTCTTTCCCTACACGACGCTCTTCCGATCT-GTGCCAGCMGCCGCGGTAA-3′) and 806R (5′-GTGACTGGAGTTCAGACGTGTGCTCTTCCGATCT-GGACTACHVGGGTWTCTAAT-3′) consisted of the Illumina (San Diego, CA, USA) paired-end adapter sequences (underlined) and 16S rRNA gene-specific sequences. The first PCR mixture for the soil DNA amplification consisted of 10 ng DNA template, 12.5 µL of 2× reaction buffer, 5 µL of 10 mM dNTP, 0.75 µL of 10 µM primers (each), 0.25 µL of KOD Fx Neo (Toyobo, Osaka, Japan), and 0.75 µL of nuclease and DNA-free water. The thermal programs of the first PCR for soil samples were as follows: 2 min at 94°C, 20 cycles of 10 s at 98°C, 30 s at 50°C, and 30 s at 68°C. For the first amplification of the plant-extracted DNA, 25 µM peptide nucleic acids, mPNA and pPNA, were applied: mPNA (N-term-GGCAAGTGTTCTTCGGA-C-term) and pPNA

(N-term-GGCTCAACCCTGGACAG-C-term), respectively (Panagene Inc., Daejeon, South Korea), to block the amplification of contaminating sequences from a eukaryotic host (Lundberg et al., 2013). PCR reaction was conducted using the following thermal programs: 2 min at 94°C, 25 cycles of 10 s at 98°C, 10 s at 78°C, 30 s at 50°C, and 30 s at 68°C. The amplified fragments were purified using Ampure magnetic beads (Beckman Coulter, Danvers, MA, USA) according to the manufacturer’s protocol. The second PCR mixture content was the same as the first PCR mixture for the soil DNA using primers provided from FASMAC Co., Ltd. (Kanagawa, Japan) and amplified using the following thermal conditions: 2 min at 94°C, 9 to 10 cycles of 10 s at 98°C, 30 s at 50°C, and 30 s at 68°C. The purification and quantification of PCR products were performed as described above. The PCR product mixture was sent to FASMAC to acquire 2 × 250 bp paired-end sequences using the MiSeq platform (Illumina).

Sequence data for the amplicons were analyzed using the QIIME2 platform, Version 2020.2 (Bokulich et al., 2018). For all paired reads, the first 20 bases of both sequences were trimmed (to remove primer sequences), and the bases after 220 were truncated (to remove low-quality sequence data). Potential amplicon sequencing errors were corrected using DADA2 to produce an ASV dataset (Callahan et al., 2016). Obtained ASVs were aligned using MAFFT (Katoh et al., 2002), and a phylogenetic tree was contracted using FastTree 2 (Price et al., 2010). Each ASV

was assigned using a naïve Bayes classifier from the SILVA 132 database (Quast et al., 2013), and then the reads for chloroplasts or mitochondria were removed. Obtained data was normalized by rarefying to 60,000 reads per samples for community analysis. The calculation of UniFrac distances, PCoA analysis for β-diversity, and Adonis PERMANOVA test for β group significance was performed using the QIIME2 platform. The Wilcoxon's rank-sum test comparing weighted UniFrac distance was performed using R software. The sequence dataset supporting the results of this study was submitted to the DNA Data Bank of Japan (https://www.ddbj.nig.ac.jp) (PRJDB11062).

Isolation, Identification, and phylogenetic analysis of bacterial isolates

The roots were homogenized with mortar and pestle in phosphate-buffered saline, and the homogenates were diluted and distributed onto isolation mediums (Data Files S1) (Bai et al., 2015; Sherwood, 1970). Plates were incubated for four days at 28°C. Colonies were picked from plates, sub-cultured on growth medium (Data Files S1), purified by streaking at least two times, and then preserved in 17% glycerol solution at −80 °C. Genomic DNA was extracted from each bacterial colony by the hot sodium hydroxide and Tris method (Truett et al., 2000). The 16S rRNA genes were amplified using 10F and 800R primer set (Table S1). The PCR mixture consisted of 1.0 µL of extracted DNA template, 12.5 µL of 2× reaction buffer, 5 µL of 10 mM dNTP, 0.75 µL of 10 µM

primers (each), 0.25 μL of KOD Fx Neo (Toyobo, Osaka, Japan), and 4.75 μL of nuclease and DNA-free water. The thermal programs of the PCR amplification were as follows: 1 min at 94°C, 30 cycles of 10 s at 98°C, 30 s at 50°C, and 1 min at 68°C. PCR products were purified using Wizard Genomic DNA Purification Kit (Promega, Madison, WI, USA) according to the manufacturer's protocol and directly sequenced by sanger sequencing using 10F primer. Identification of individual bacteria isolates at the genus level was performed by BLAST search using DDBJ database. To obtain full-length 16S rRNA gene sequence of bacterial isolates, PCR products amplified as described above with 10F and 1500R primer set (Table S1) were cloned into pCR4-TOPO vector using TOPO TA Cloning Kit for sequencing (Invitrogen), and sequenced using M13F and M13R primers (Table S1) according to the manufacturer's protocol. The resulting near-complete 16S rRNA gene sequences were aligned with the Clustal Omega program. The 16S rRNA gene sequence data for the type strains of the family *Methyulobacteriaceae* were retrieved from the GenBank database. Phylogenetic trees were constructed by the neighbor-joining method using MEGA version 7.0 (Kumar et al ., 2016).

Whole genome sequencing of *Methylobacteriaceae* isolates

The genomic DNA of *Methylobacteriaceae* isolates was extracted as described by Hahn and Hennecke with a modification (Hahn and Hennecke, 1984). Briefly, the cells were lysed by the

addition of 20 mg lysozyme, 10% sodium dodecyl sulfate, and proteinase K (20 mg mL^{-1}) and incubated for 1 h at 37°C. The cell lysate was forced through a syringe (21G×1-1/2″ RB; Terumo Corporation, Tokyo, Japan) and extracted with phenol and chloroform. The genomic DNA was dissolved in TE buffer and kept at 4°C. For SMRTbell library preparation, each genomic DNA was fragmented at 20 kbp using a Megaruptor2 [Diagenode, Seraing (Ougrée), Belgium], and the library was constructed using SMRTbell Express Template Prep Kit 2.0 according to the manufacturer's protocol (Pacific Biosciences, Menlo Park, CA, USA). The barcodes were attached to each fragmented genome, and the samples were pooled and cut off at 15 kbp using the BluePippin size selection system (Sage Science, Cummings Center Beverly, MA, USA). The genomic library was sequenced on a single PacBio sequel II system 2.0 cell. Genomes were assembled with HGAP4 via SMRTlink (version 8.0.0) using the specified genome sizes. The sequence dataset was submitted to the DNA Data Bank of Japan (https://www.ddbj.nig.ac.jp) (PRJDB11299).

Reference

Bai, Y., Müller, D. B., Srinivas, G., Garrido-Oter, R., Potthoff, E., Rott, M., et al. (2015). Functional overlap of the *Arabidopsis* leaf and root microbiota. *Nature,* **528** (7582), 364-369. doi:10.1038/nature16192

Bokulich, N. A., Kaehler, B. D., Rideout, J. R., Dillon, M., Bolyen, E., Knight, R., et al. (2018). Optimizing taxonomic classification of marker-gene amplicon sequences with QIIME 2's q2-feature classifier plugin. *Microbiome,* **6** (1), 90. doi:10.1186/s40168-018-0470-z

Hahn, M., and Hennecke, H. (1984). Localized mutagenesis in *Rhizobium japonicum*. *Mol Gene Genet,* **193**, 46-52.

Katoh, K., Misawa, K., Kuma, K. i., and Miyata, T. (2002). MAFFT: a novel method for rapid multiple sequence alignment based on fast Fourier transform. *Nucleic Acids Research,* **30** (14), 3059 3066. doi:10.1093/nar/gkf436

Kumar, S., Stecher, G., and Tamura, K. (2016). MEGA7: Molecular evolutionary genetics analysis Version 7.0 for bigger datasets. *Mol Biol Evol,* **33** (7), 1870-1874. doi:10.1093/molbev/msw054

Lundberg, D. S., Yourstone, S., Mieczkowski, P., Jones, C. D., and Dangl, J. L. (2013). Practical innovations for high-throughput amplicon sequencing. *Nat Methods,* **10** (10), 999-1002. doi:10.1038/nmeth.2634

Price, M. N., Dehal, P. S., and Arkin, A. P. (2010). FastTree 2 – Approximately Maximum

Likelihood Trees for Large Alignments. *PLoS One,* **5** (3), e9490. doi:10.1371/journal.pone.0009490

Quast, C., Pruesse, E., Yilmaz, P., Gerken, J., Schweer, T., Yarza, P., et al. (2013). The SILVA ribosomal RNA gene database project: improved data processing and web-based tools. *Nucleic Acids Res,* **41**(Database issue), D590-596. doi:10.1093/nar/gks1219

Sherwood, M. T. (1970). Improved synthetic medium for the growth of *Rhizobium*. *J Appl Bacteriol,* **33** (4), 708-713. doi:10.1111/j.1365-2672.1970.tb02253.x

Truett, G. E., Heeger, P., Mynatt, R. L., Truett, A. A., Walker, J. A., and Warman, M. L. (2000). Preparation of PCR-quality mouse genomic DNA with hot sodium hydroxide and tris (HotSHOT). *Biotechniques,* **29** (1), 52, 54. doi:10.2144/00291bm09
